# 

**DOI:** 10.16910/jemr.10.5.8

**Published:** 2017-12-13

**Authors:** Jonathan Allsop, Rob Gray, Heinrich H. Bülthoff, Lewis Chuang

**Affiliations:** Vision and Eye Research Unit, Anglia Ruskin University, UK; Human Systems Engineering Department, Arizona State University, USA; Department of Human Perception Cognition and Action. Max Planck Institute for Biological Cybernetics, Germany

**Keywords:** instruments, anxiety, cognitive load, eye tracking, heart rate, entropy, attention

## Abstract

In this study, we demonstrate the effects of anxiety and cognitive load on eye movement planning in an instrument flight task adhering to a single-sensor-single-indicator data visualisation design philosophy. The task was performed in neutral and anxiety conditions, while a low or high cognitive load, auditory n-back task was also performed. Cognitive load led to a reduction in the number of transitions between instruments, and impaired task performance. Changes in self-reported anxiety between the neutral and anxiety conditions positively correlated with changes in the randomness of eye movements between instruments, but only when cognitive load was high. Taken together, the results suggest that both cognitive load and anxiety impact gaze behavior, and that these effects should be explored when designing data visualization displays

## Introduction

Many critical-safety domains require operators to continuously monitor and process a large number of variables. In this digital age, it might seem surprising that many of the interfaces in complex environments (e.g., flight cockpits, air traffic control centers, power plants) continue to observe a “single-sensor-single-indicator” (SSSI; as termed by Goodstein [ 18 ]) design guideline for data visualization—whereby low-level readings from environment sensors are directly and independently communicated to the operator via dedicated instruments. The rationale for SSSI’s continued use is that separate information channels allows for transparency and for individual data elements to be flexibly combined by trained operators to generate appropriate responses for all possible operational purposes, even for situations that might not be anticipated by the interface designers [ 14 ]. Moreover, complex systems are often composed of multiple sub-systems that are highly-coupled (see Methods for an example; a fixed wing landing task). Thus, the display of individual data elements not only communicates the data per se, but also allows the operator to monitor the changing relationship between sub-groups of multiple variables.

The limitations to SSSI are intuitively apparent. A busy array of instruments requires operators to sequentially seek out data with eye-movements, which then has to be integrated. In other words, visual scanning and the cognitive load on working memory are believed to place a burden on operators, which might be obviated with information visualization designs that supported decision-making instead of “data availability” [ 46 ]. This belief has motivated displays, such as those termed “ecological interface designs”, that seek to maximize “information extraction” by exploiting our seemingly limitless capacity for pattern recognition [ 6, 36 ]. In an early example, the data of 100 sensor values of a nuclear power plant, which hitherto had to be individually monitored, were mapped into a single centralized octagon display [ 47 ]. In this example, growing distortions in the octagon’s symmetry indicated a developing abnormality. To fully appreciate (and justify the practical implementation of) ecological interface designs, especially in safety-critical domains, it is necessary to demonstrate that relying on SSSIs is indeed effortful and vulnerable to variable human factors.

Operators often utilize such displays in demanding and stressful situations. Stress is an interactive process whereby a demand is placed on an operator and the response is determined by a combination of the details and appraisal of the stressor, along with perceived coping resources. Where stressors outweigh perceived coping abilities, state anxiety is likely to be invoked, which is an acute negative emotion related to a specific event and is characterized by “consciously perceived feelings of tension and apprehension” ([ 34 ], p. 17). Relatedly, trait anxiety is a general disposition where individuals respond to stressful situations with high levels of state anxiety [ 45 ]. In the present paper, we are specifically interested in examining the effects of working memory load and state anxiety during SSSI use.

Visual scanning behavior is influenced by multiple factors, which interact to determine our ability to acquire just-in-time and task-relevant information from the environment. Attentional control theory (ACT; [ 16 ]) offers a comprehensive framework that explicitly considers the relationships between attention, the working memory system, and anxiety. Thus, it can serve to help us understand how access to visual displays with more than one region of interest might be susceptible to user states. ACT is based around previously delineated attentional sub-systems, a goal-directed system and a stimulus-driven system (see [ 10 ]). The goal-directed system controls attention based on current or future goals, past experience, and predictions. Whereas the stimulus driven system directs attention based on the saliency and expectancy of sensory events. In the context of aviation, purposeful eye movements across different instruments are associated with higher proficiency and random eye movements with worse proficiency [ 8 ].

ACT postulates that anxiety can lead to a modification in the balance between the attentional sub-systems presented previously. Specifically, it is suggested that anxiety leads to decreased prioritisation of the goal-directed system, with the stimulus driven system gaining increased control over the allocation of attention. This change in prioritisation decreases the likelihood of attention being efficiently directed toward goal-relevant information. The reprioritisation is underpinned, according to ACT, by anxiety-induced changes to the functioning of specific (see [ 25 ]) working memory functions, namely: inhibition, shifting and updating. It is predicted that anxiety can lead to reduced efficiency in inhibiting inappropriate prepotent responses, and maintaining attention on task relevant information. It is also predicted that anxiety can impair the ability to switch between tasks, and update and monitor the information in working memory. Thus, in SSSI based tasks, impairments in the ability to seek task-relevant information, switch between sub-tasks, and monitor and updated information in working memory, seem very likely to be detrimental for performance.

Changes to gaze behavior have been identified in tasks performed under anxious conditions, with results providing support for ACT’s predicted influence of anxiety on attentional control [ 3, 7, 40 ]. Anxiety has been shown to increase the frequency of fixations on goal-irrelevant stimuli [ 41 ] and reduce the duration of ordinarily long target-focused fixations [ 7, 26 ]. In the context of SSSI based tasks, Allsop and Gray [ 1 ] successfully used ego-threatening instructions and monetary incentives to induce anxiety in participants with extensive practice in instrument scanning for performing a flight landing task. Anxiety led to decreased percentage dwell time on the instruments, and increased time on the external world. Scanning entropy, which is indicative of the randomness of visual scanning, also increased. Interestingly, changes in anxiety positively correlated with changes in scanning randomness. In a partially analogous context, Vine and colleagues [ 37 ] investigated the effects of stress on gaze behavior in commercial pilots as they encountered a simulated emergency situation, during an important periodic proficiency exam. Perceiving the exam to be more threatening (defined by subjectively rating the task to be demanding, along with low coping evaluations) was associated with higher search rates and more fixations on unimportant instrument locations. Such evaluations were also marginally related to increases in scanning entropy.

Turning to the effects of cognitive load on gaze behavior in tasks requiring visual scanning. In a hazard perception task, cognitive load has been shown to lead to a longer duration to first fixate on the hazard and, interestingly, also reduce the average hazard fixation durations for individuals with lower working memory capacities [ 43 ]. Cognitive load has also been shown to lead to more spatially concentrated gaze behavior during real-world driving [ 30 ]. With relation to SSSI displays, cognitive load has been shown to increase the average dwell time on instruments [ 35 ].

Cognitive load may exacerbate the effects of anxiety on gaze behavior, as, like other interference theories of anxiety [ 33 ], ACT suggests that anxiety consumes a limited pool of working memory resources. Therefore, when working memory demands converge on working memory limits, anxiety-induced attentional changes may be more likely to occur [ 4 ]. For instance, individuals with lower working memory capacities exhibit stronger negative relationships between anxiety and simple, processpure, measures of attentional control [ 31, 21 ]. Of more central interest, studies have also directly manipulated demands on working memory, and investigated its interaction with anxiety. Increasing cognitive load has been shown to compound the effects of anxiety in simple, tests measuring specific aspects of attentional control [ 5, 28 ].

A limited number of studies have examined the combined influence of anxiety and working memory on gaze behavior [ 27, 39 ]. Findings from these studies are less homogenous. Some studies have not found an interactive effect of anxiety, with cognitive load [ 27 ] or working memory capacity [ 44 ], whereas others have [ 39 ].

In sum, the present paper aims to elucidate the influence of anxiety and cognitive load on information seeking behavior during a task adhering to SSSI visualisation design philosophy [ 12 ], namely an instrument flight task.

## Methods

### Apparatus

**Figure 1.**
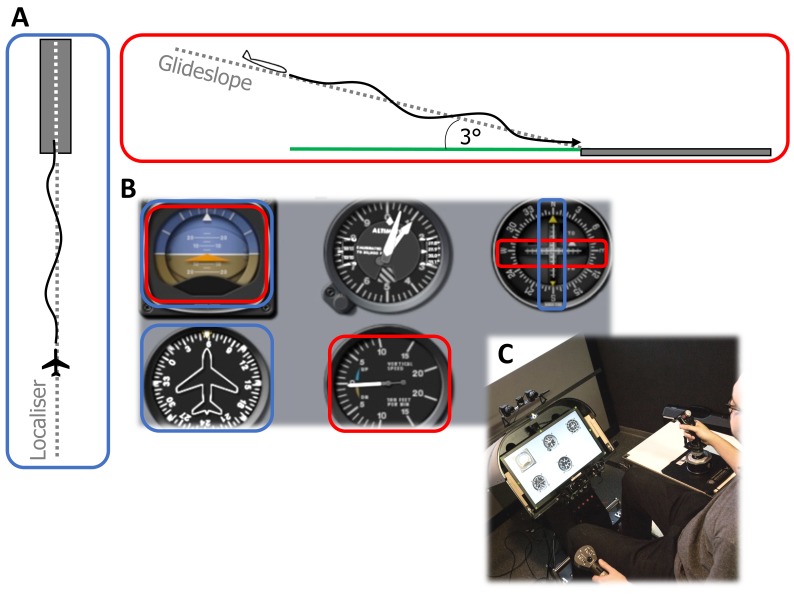
A: Schematic representation of the instrument landing task from a side-on (red outline) and top-down (blue outline) view (not to scale). Participants attempt to follow the ideal vertical (glideslope) and lateral (localiser) paths using the cockpit instruments. B: Layout of the heads-down instrument panel showing, from top-left, in a clockwise direction: attitude direction indicator, altimeter, instrument landing course deviation indicator, vertical speed indicator, heading indicator. The instruments required to track the ideal: vertical path are outlined in red, and lateral path are outlined in blue. C: Photograph of the experimental setup showing the heads-down instrument panel, back-projection screen, control devices and eye-tracking cameras

A Thrustmaster HOTAS Warthog joystick (Guillemot, Montreal, Canada) was used to control the roll and pitch axis of a Cirrus Vision SPF50, simulated within X-Plane version 10 (Laminar Research). The landing gear and flaps were extended, with auto throttles set to maintain airspeed at 100 knots (51.4 ms^-1^). Flight data was recorded at a rate of 52 Hz. The virtual world was displayed on the upperhalf (0.96m) of a large screen (2.20 x 1.92 m; 1400 x 1050 pixels) using a back-projection system (Christie Mirage S+3K DLP; 101 Hz), while the rest of this screen was set to black. A ‘heads-down’ electromechanical-style instrument panel (see Figure 1) was displayed on a TFT monitor (45 x 25 cm; 1600 x 1900 pixels). This instrument panel displayed five instruments in two rows. The attitude indicator (AI), altimeter (Alt) and instrument landing system course deviation indicator (ILS) were displayed on the top row, while the heading indicator (Hdg) and vertical speed indicator (VSI) were displayed on the second row. The projection screen and heads-down monitor were at 1.8 and 1.0 m viewing distances, respectively. A remote eye tracking (FaceLAB, Seeing Machines) system recorded eyemovements (precision < 1.0 °) at 60 Hz. The auditory stimuli for the cognitive load task were delivered using closed ear headphones (Beyerdynamic DT770 Pro), and participants responded using a push-button on a custom-made USB ‘collective’ joystick.

### Task

The objective of the task was to land the aircraft accurately by following an ideal approach path. This ideal path is comprised of both lateral and vertical components, termed the localiser and glideslope, respectively. The localizer is simply an extension of the runway centerline. The glideslope component is a 2D plane extending upwards from the end of the runway at an angle of 3º. The aircraft was positioned 6 nautical miles (11.11 km) from the runway at the start of each trial and orientated (heading, roll and pitch) for a perfect approach. All trials were performed in low visibility, instrument meteorological conditions (IMC), with visibility set to 1.2km. This low visibility meant that participants were required to use cockpit instruments to follow the ideal approach path. Wind speed was set to 20 knots (10.3 ms^-1^), but the direction was varied based on the experimental phase, as described in more detail in the procedure section below. Numerical and graphical performance feedback was displayed on the back projection screen after each trial. Numerical feedback consisted of the vertical and lateral performance errors (detailed in the measures section). Graphical feedback consisted of a graphical representation of the ideal vertical and lateral paths compared against the participants’ actual paths.

### Participants

Sixteen participants (5 Female; mean age = 26.6, SD = 3.8) completed the study. All participants reported normal or corrected vision, were right handed and had no previous real or simulated fixed-wing flight experience. Participants were paid for their participation at a rate of 8 euros per hour. A university ethics committee granted ethical approval for the study and all participants provided informed consent.

### Measures

#### Cognitive Anxiety

Cognitive state anxiety was measured using the cognitive anxiety subscale from the Competitive State Anxiety Inventory 2-revised [ 11 ]. This subscale contains five items, with an example item being “I’m concerned about performing poorly”. After each landing in the experimental phase, participants were asked to rate on a four point scale ranging from 1 (not at all) to 4 (very much so), whether each item corresponded to how they thought of felt during the landing. Item responses were averaged and then multiplied by 10 in accordance with Cox et al., [ 11 ].

#### Heart Rate

A chest-strap heart rate (Garmin Model HRM1G) was used to provide physiological evidence of the effectiveness of the anxiety manipulation. The strap was moistened and positioned on the lower-mid thorax. Data was transmitted wirelessly to a laptop, which recorded data at 1Hz throughout each experimental trial. Heart rate was then averaged for each trial.

#### Performance

Root mean square error (RMSE) of the vertical deviation from the ideal glideslope was used as the flight performance metric, similar to previous studies [ 1, 17 ]. This was derived from the recorded ILS instrument data, with the unit of measurement therefore being in dots. One glideslope dot represents 0.28° error in X-Plane. Vertical and lateral RMSE was displayed after each trial, with one lateral dot equaling 1.5° error.

#### Gaze Behavior

Horizontal and vertical screen coordinates (corresponding to eye-gaze location) for the external world and instrument panel were recorded by the eye-tracker software (Facelab, Version 5; Seeing Machines). A dispersion threshold identification algorithm (c.f., [ 32 ]) was used to convert these coordinates into fixations, with the minimum fixation threshold being set to 150ms in accordance with previous research [ 20 ]. Fixations were then assigned to six areas of interest (AOIs) based on their on-screen coordinates and were confirmed manually. These AOIs were: external view, attitude indicator, altimeter, instrument landing course deviation indicator, heading indicator and vertical speed indicator. Fixations were converted into dwells to provide dwell frequencies and durations. To examine general changes in attentional allocation, individual AOIs were subsumed within two AOIs, namely: external world and instrument panel (which included of all instrument panel AOIs). Percentage dwell time on this AOI and the external world AOI were used as dependent measures.

The randomness of scanning behavior, termed Scanning entropy, was calculated in an identical manner to Allsop and Gray [ 1 ] using Ellis & Stark’s [ 15 ] methodology. Higher values on this metric indicate more random scanning behavior, whereas lower values indicate more predictable scanning behavior.

### Procedure

Participants visited the lab on two occasions separated by a stipulated maximum interval of one week, with each visit lasting approximately two hours. The experiment was split into two phases: an acquisition phase, which developed the participants’ ability to perform the task; and an experimental phase, where both cognitive anxiety and cognitive load were manipulated.

#### Acquisition phase

Participants completed 22 acquisition trials during this phase, with first 13 trials being completed in the first lab visit and 9 in the second. To ensure that the cockpit instruments were required to successfully perform the task, as opposed to simply adopting a proceduralised method, the simulated wind was set randomly for the first 19 acquisition trials. Specifically, the wind direction was randomly chosen from one of 4 angles: 20º, 160º, 200º and 340º; where 0º represents a direct headwind. For the final three acquisition trials, wind was set to 160º.

At the beginning of the first lab visit, participants provided informed consent, and eye-tracker compatibility was checked by performing a calibration. Participants were then given an information sheet with details of the flight task and cockpit instruments. The experimenter then verbally explained the task and the cockpit instruments. In order to aid motivation and acquisition of the task, a recommended order for fixating on the instruments (based on recommendations by a certified flight instructor) was explained. The recommended order was as follows: ILS to AI, AI to HDG, HDG to VSI and VSI to ILS. The experimenter then demonstrated the landing task to the participant. Afterwards, participants were allowed a 5 minute free-flight to acclimatize to the controls, cockpit instruments and simulator. Participants then completed the acquisition trials for the first visit. For the first three of these trials, the performance feedback was supplemented by verbal feedback by the experimenter, due to the initial complexity of the task. At the start of the second session, the heart rate monitor was positioned and the eye-tracker was calibrated. The participant then completed the remaining 9 acquisition trials.

#### Experimental phase

Cognitive anxiety and cognitive load were manipulated in this experimental phase. A 2 cognitive load (Low, High) x 2 anxiety condition (Neutral, Anxiety) within-subjects design was employed. Participants therefore performed a total of 4 trials in this phase. The ordering of anxiety trials was counterbalanced across participants – with half performing anxiety trials first while the other half performed neutral trials first. The ordering of cognitive load conditions was also counterbalanced across participants, the ordering was the same in neutral and anxiety conditions. For all trials wind direction was set to 160º.

At the start of this phase participants were instructed that for the remaining trials they would be required to perform an auditory task at the same time as performing a landing task. It was emphasised that equal importance should be placed on both tasks. Four familiarisation attempts at the cognitive task (one low-load, three high-load) were performed without flying (these were not recorded). The experimental trials were then performed. Flight data, heart rate and gaze behavior were measured at the start of the trial and saved upon trial completion, at which point cognitive anxiety was measured. Participants were fully debriefed on the nature of the study at the end of all the experimental trials.

##### Cognitive load manipulation

An auditory n-back task [ 24 ] was used to manipulate cognitive load. A series of auditory stimuli were presented sequentially at an interstimulus interval of two seconds [ 23 ]. For each stimulus, the participant was instructed to respond as quickly and accurately as possible if it was a target. In the low load condition, n was set to 0, and participants simply listened for one specific, pre-disclosed, target stimulus. In the high cognitive load condition, n was set to 2, where a stimulus is a target only when it is the same as two stimuli before. The auditory stimuli consisted of a pool of 14 consonants. 25% of stimuli were targets for both conditions. Reaction time and percentage accuracy were measured. Incorrect responses were excluded from reaction time analyses, as were responses of less than 300ms (no responses fell below this threshold).

##### Anxiety manipulation

Anxiety was manipulated using a combination of monetary incentives and ego-threatening instructions, in a nearly identical manner to [ 1 ]. Similar manipulations have been shown to be successfully increase anxiety in a number of other experiments [ 9, 39 ]. For neutral, lowanxiety trials the instruction to participants was simply to “perform the best they can”. For high-anxiety trials, the manipulation consisted of three steps.

Firstly, participants were informed immediately prior to commencing the trials that they could now win 50 euros based on the combined performance over the next two trials. Specifically, they were informed that they would be ranked against everyone else taking part, and that the person with the lowest RMSE (best performance), would be rewarded. A leaderboard was revealed and participants were told that the leaderboard would be e-mailed out to participants at the end of data collection. Secondly, a video camera (Sony DCR-TRV890E) was overtly set-up on a tripod located behind the participant. Participants were informed that both trials would be video recorded for potential use in upcoming conference presentations and lectures, and that their video would be used if their performance was significantly below average. Thirdly, participants were told that they would be flying in an online virtual ( www.vatsim.net ), and the experimenter loaded a custommade program that allowed the experimenter to enter a mock log-in and connection to be made. Upon ‘loggingin’, the program opened a world-mapping program (Marble, Version 1.6) which was edited to show a top-down view of the airport and surrounding area. This area was populated with other aircraft and extended trail histories. Upon completion of all the experimental trials, participants were debriefed on the true nature and reasoning behind this manipulation.

### Statistical Analyses

Cognitive anxiety, heart rate, n-back percentage correct, n-back reaction time, Glideslope RMSE, transition frequency and scanning entropy were analysed using separate 2 anxiety condition (neutral conditions, anxiety conditions) x 2 cognitive load (low cognitive load, high cognitive load) repeated measures ANOVAs. The effects of anxiety and cognitive load on attentional allocation were examined by submitting percentage dwell time data to a 2 anxiety condition neutral, anxiety) x 2 cognitive load (low, high) x 2 AOI (external, instruments) repeated measures ANOVA. Significant effects were analysed using Tukey’s HSD post hoc procedures (p < .05).

In line with our expectations and previous research [ 19, 38 ], analyses were performed in order to examine whether an individual’s response to the anxiety manipulation may be related to scanning entropy, and also whether cognitive load may moderate this relationship. Difference scores between neutral conditions and anxiety conditions for both low- and high cognitive load conditions, were created for the cognitive anxiety, entropy and performance variables. This procedure is similar to within-subject mediation and moderation procedures outlined by [ 22 ]. Three linear regressions were then performed.

The simple overall relationship between change in entropy and anxiety, independent of any potential moderation effects, was investigated by collapsing the high and low cognitive load data. Change in entropy was then regressed onto change in cognitive anxiety. To investigate whether cognitive load may moderate any relationship between change in cognitive anxiety and change in entropy, two separate linear regressions were then performed for data from the low and high cognitive load conditions. Raghunathan, Rosenthal, & Rubin's [ 29 ] modification of the Pearson’s correlation coefficient statistic was then used to formally compare whether there was a difference in the relationship between change in cognitive anxiety and change in entropy based on cognitive load.

## Results

The cognitive anxiety, heart rate, n-back and performance results will be presented first. Then the following eye movement analyses will be presented: percentage dwell time, transition frequency and scanning entropy.

### Cognitive Anxiety

**Figure 2.**
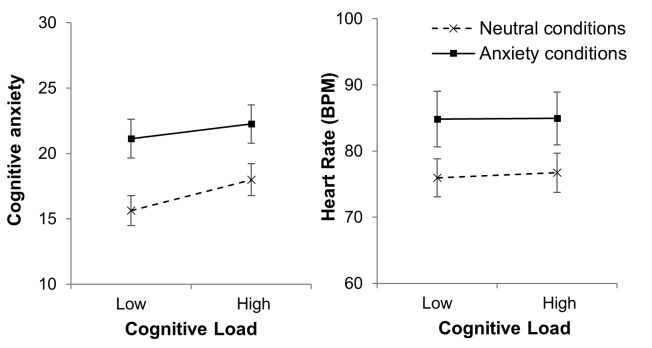
Mean (S.E.M) cognitive anxiety (left panel) and heart rate (right panel) plotted as a function of cognitive load in neutral (dashed line) and anxiety (solid line) conditions.

Mean cognitive anxiety data is displayed in figure 2 (left panel). Significant main effects for both anxiety condition, F(1,15) = 10.19, p = .006, η^2^_p_ = .41 and cognitive load, F(1,15) = 6.62, p = .02, η^2^_p_ = .31 were found. There was no significant interaction between Anxiety condition and Cognitive load, F(1,15) = 1.62, p = .22, η^2^_p_ = .10. Breakdown of the main effects revealed that cognitive anxiety was higher in the anxiety condition than the neutral condition, and higher in the high cognitive load condition than the low load condition.

**Figure 3:**
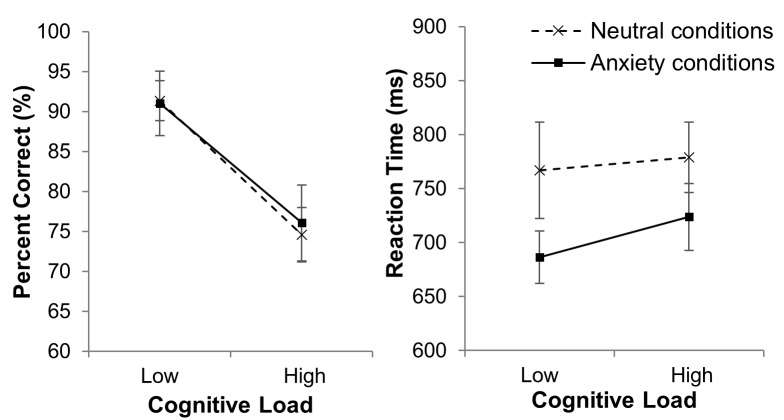
Mean (S.E.M) n-back percent correct (left panel) and reaction time (right panel) plotted as a function of cognitive load in neutral (dashed line) and anxiety (solid line) conditions.

### Heart Rate

Mean heart rate data is displayed in figure 2 (right panel). A significant main effect for anxiety condition was found, F(1,15) = 18.07, p = .001, η^2^_p_ = .55. The main effect for cognitive load was non-significant, F(1,15) = .36, p = .56, η^2^_p_ = .02 and a the interaction between Anxiety condition and cognitive load was non-significant, F(1,15) = .26, p = .62, η^2^_p_ = .02. Heart rate was higher in the anxiety conditions.

### N-back Task

#### Percentage Correct

Mean percentage correct data is displayed in figure 3 (left panel). The ANOVA conducted on these data revealed a non-significant main effect for anxiety condition, F(1,13) = .13, p = .73, η^2^_p_ = .01, a significant main effect for cognitive load, F(1,13) = 49.59, p < .001, η^2^_p_ = .77, and a non-significant interaction between Anxiety condition and Cognitive load, F(1,13) = .001, p = .98, η^2^_p_ = .01. Less correct n-back responses were made in high cognitive load conditions.

#### Reaction Time

Mean reaction time data is displayed in figure 3 (right panel). The analysis revealed a significant main effect for anxiety condition, F(1,13) = 7.64, p = .016, η^2^_p_ = .19, a non-significant main effect for cognitive load, F(1,13) = 1.52, p = .24, η^2^_p_ = .19, and a non-significant interaction between anxiety condition and cognitive load, F(1,13) = .35, p = .56, η^2^_p_ = .01. Reaction time was shorter in anxiety conditions.

### Performance

The analysis of glideslope RMSE data (See table 1) revealed a non-significant main effect for anxiety condition, F(1,15) = 0.16, p = .90, η^2^_p_ = .001, a significant main effect for cognitive load, F(1,15) = 4.62, p = .048, η^2^_p_ = .24, and a non-significant interaction between anxiety and cognitive load conditions, F(1,15) = .15, p = .70, η^2^_p_ = .01. Examination of the main effect for cognitive load showed that performance deteriorated in high cognitive load conditions. In sum, performance was maintained in anxious conditions, but deteriorated when cognitive load was high.

### Gaze Behavior

#### Percentage dwell time

**Figure 4:**
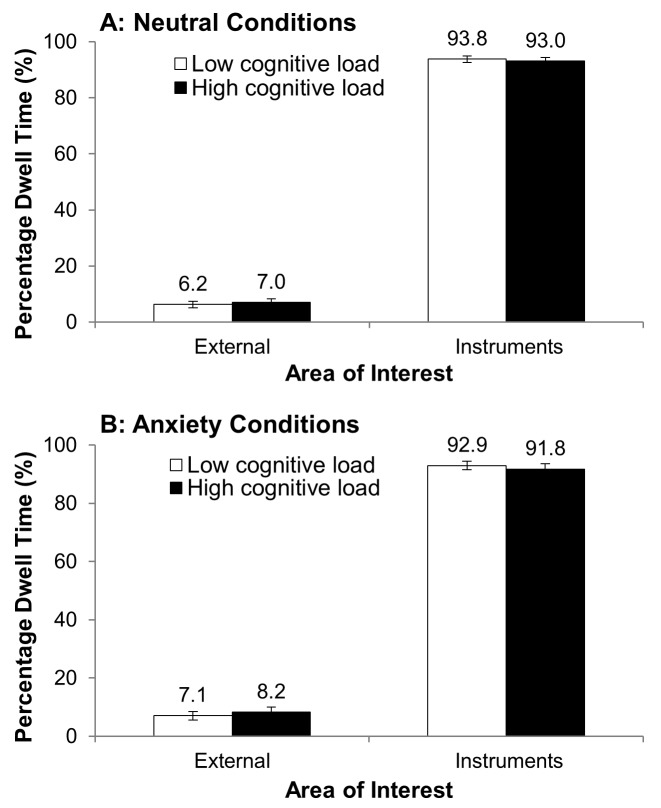
Mean (S.E.M) percentage dwell time on the external world and the generalized instrument panel AOIs, in the neutral conditions (Panel A) and anxiety conditions (Panel B) in low cognitive load and high cognitive load conditions.

Figure 4 shows the mean percentage dwell time data. A marginally significant interaction between anxiety condition and AOI was revealed, F(1,15) = 4.15, p = .06, η^2^_p_ = .22, and a non-significant interaction between cognitive load and AOI, F(1,15) = 1.35, p = .26, η^2^_p_ = .08. The anxiety condition and AOI interaction was explored by examination of the mean data. This shows a tendency in anxiety conditions for percentage dwell time on the outside world to be higher, and percentage dwell time on the instruments to be lower, when compared to neutral conditions. The analysis revealed a non-significant Anxiety condition x Cognitive load x AOI interaction, F(1,15) = .236, p = .63, η^2^_p_ = .02. This suggests that cognitive load did not moderate the tendency to look towards the outside world in anxiety conditions. All other interactions were non-significant (p’s > .2).

#### Transition Frequency

Table 1 shows the transition frequency data. The ANOVA conducted on these data revealed a non-significant main effect for anxiety condition, F(1,15) =.05, p = .82, η^2^_p_ = .003, a significant main effect for cognitive load, F(1,15) = 22.78, p < .001, η^2^_p_ = .60, and a non-significant interaction between anxiety condition and cognitive load, F(1,15) = .41, p = .53, η^2^_p_ = .03. Transitions between areas of interest were less frequent in high cognitive load conditions than low cognitive load conditions.

**Table 1.** Mean (SD) Glideslope RMSE, transition frequency, and scanning entropy in experimental conditions

	Neutral Conditions			Anxiety Conditions	
Measure	Low cognitive load	High cognitive load		Low cognitive load	High cognitive load
Glideslope RMSE (dots)	0.46 (0.27)	0.53 (0.35)		0.44 (0.23)	0.53 (0.26)
Transition frequency	187.81 (27.45)	169.63 (36.53)		188.88 (33.68)	166.50 (34.59)
Scanning entropy	1.38 (0.18)	1.41 (0.18)		1.44 (0.20)	1.40 (0.19)

#### Scanning Entropy

Mean scanning entropy data is displayed in Table 1. To reiterate, higher scanning entropy values indicates that eye-movements between instruments were more random, while lower scanning entropy values indicates more predictable and, hence, planned scanning behavior. The analysis revealed a non-significant main effect for anxiety condition, F(1,15) = .30, p = .59, η^2^_p_ = .02, a non-significant main effect for cognitive load, F(1,15) =.23, p = .88, η^2^_p_ = .002, and a non-significant Anxiety condition x Cognitive load interaction, F(1,15) = 2.27, p = .15, η^2^_p_ = .13.

#### Individual Responses to the Anxiety Manipulation

**Figure 5.**
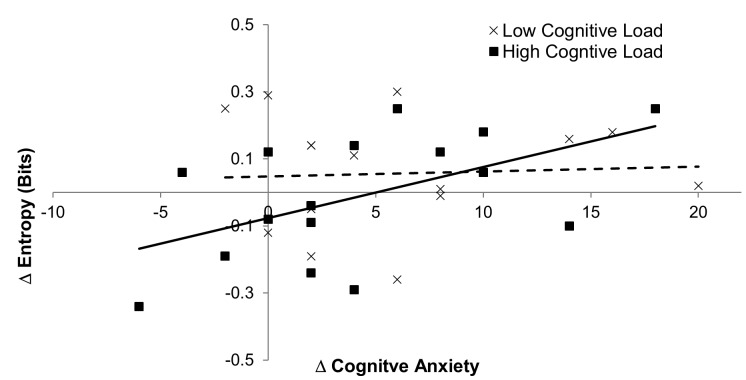
Scatter plot with linear regression lines showing the relationship between change in cognitive anxiety and change in entropy, in low (dashed line) and high (solid line) cognitive load conditions

When data was collapsed across cognitive load, change in cognitive anxiety was a marginally significant predictor of change in scanning entropy, b = .009, 95% CI [-.001, .19], t = 1.867, p = .07, explaining 10% of the variance in entropy scores. The potential moderating role of cognitive load was then examined (see Figure 5). For low cognitive load conditions, change in cognitive anxiety did not predict change in scanning entropy, b = .002, 95% CI [-.013, .17], t = 0.23, p = .82 and did not explain a significant proportion of the variance in change in entropy scores, R^2^ = .004. Interestingly however, when cognitive load was high, change in cognitive anxiety was a significant predictor of change in scanning entropy, b = .015, 95% CI [.001, .03], t = 2.32, p = .036, explaining 28% of the variance^1^. There was also a significant difference between the correlation coefficients, z = 1.72, p = .028. Together, these results suggest that cognitive load moderated the relationship between change in cognitive anxiety and change in scanning entropy, with the positive relationship being stronger when cognitive load was high, than when cognitive load was low.

**Figure 6.**
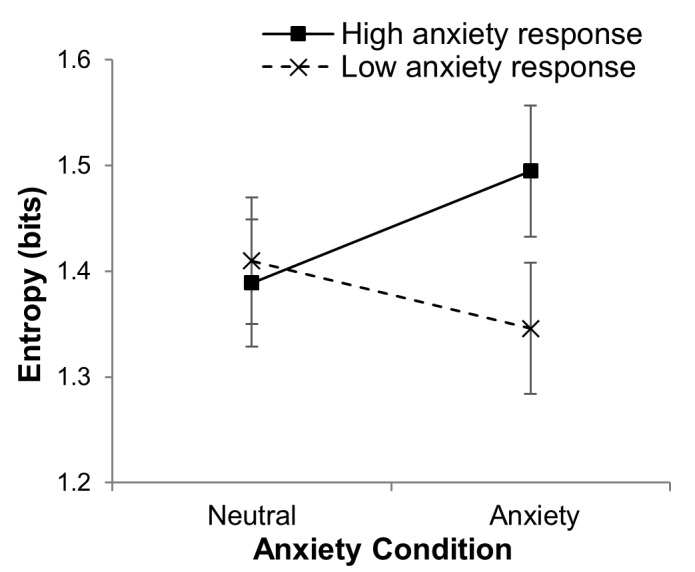
Mean scanning entropy (S.E.M) plotted as a function of anxiety condition for the high (solid line) and low (dashed line) anxiety manipulation response groups

A supplementary median-split approach is presented to more concretely illustrate that an individual’s response to the anxiety manipulation critically influenced the impact of the anxiety condition on scanning entropy. Specifically, participants were categorized into high and low anxiety manipulation response groups, based on their difference score between averaged (across low and high cognitive load conditions) cognitive anxiety ratings in the neutral and anxiety conditions. An independent sample t-test confirmed that these two groups were significantly different t(14) = -4.39, p = .001 (difference scores: high response group = 9.38 ±5.3; low response group = 0.38 ± 2.33). A 2 manipulation response (low anxiety manipulation response, high anxiety manipulation response) x 2 anxiety condition (neutral, anxiety) x 2 cognitive load ANOVA with repeated measures on the last two factors revealed a significant interaction between manipulation response and anxiety condition, F(1,14) = 6.84, p = .02, η^2^_p_ = .33. The nature of this interaction is shown in Figure 6, with entropy being higher in anxiety conditions for the high manipulation response group in comparison to the low response group.

## Discussion

This study aimed to demonstrate the effects of anxiety and cognitive load on information seeking behavior in a control scenario adhering to the SSSI visualisation design philosophy. It was framed within attentional control theory (ACT; Eysenck et al., [ 16 ]), which provides an account for how anxiety and the working memory system interacts to influence the control of attention. Participants first undertook training to perform an instrument landing task where information had to be acquired from discrete cockpit instruments in order to complete the task accurately. Then, during testing, both anxiety and cognitive load were manipulated.

The effectiveness of the anxiety manipulation was validated by increases in self-reported cognitive anxiety and objective heart rate between the neutral and anxiety conditions. This offers support for the use of evaluative instructions and monetary incentives for this purpose, with the average increase in heart rate being comparable to results from previous studies using similar manipulations (e.g., Cooke et al., [ 9 ]; Moore et al.,[ 26 ]). Self-reported anxiety was also higher in the high versus low cognitive load conditions, suggesting that participants had more concerns over their ability to perform the task under high cognitive load.

Cognitive load was successfully manipulated using an auditory n-back task, with more incorrect responses in high, compared to low, cognitive load conditions. Importantly, response times remained the same across these two conditions, which quells concerns over a speed-accuracy tradeoff. Interestingly, anxiety led to decreased reaction time, while accuracy was maintained. A likely explanation for this finding is that anxiety was accompanied by a compensatory increase in effort and auxiliary processing resources [ 16 ]. This behavioral finding is supported by increases in self-reported effort that have been previously shown to accompany anxiety [ 9, 42 ], and offers an explanation for flight task performance being maintained in anxious conditions in the current study. This is also in line with ACT, which makes an important distinction between performance effectiveness and processing efficiency. Performance effectiveness is the overall performance outcome, whereas processing efficiency refers to the effort or resources invested in order to achieve a performance outcome, with processing efficiency more readily being impacted than performance effectiveness. In the present study, maintaining flight performance in anxious conditions was achieved at the cost of reduced processing efficiency, as evidenced by both reduced n-back response times (i.e., more resources) and the anxiety-induced changes to gaze behavior detailed below.

Gaze behavior was significantly impacted by cognitive load, with a reduction in the number of transitions between areas of interest (e.g., instruments) being found. This indirectly supports previous studies showing cognitive load to increase average dwell time on SSSIs [ 35 ] and decrease the variability of gaze location [ 31 ], as both these gaze changes will consequently likely lead to less transitions. It is probable that this decrease in transition frequency led to instruments being inadequately sampled and thus was responsible for the observed impairment of flight task performance in high cognitive load conditions.

In anxious conditions, there was a tendency for participants to look more towards the outside world as opposed to the instrument panel. Although this finding was marginally significant (p = .06), it is qualitatively similar to Allsop & Gray’s [ 1 ] findings. In accordance with ACT and previous work, we suggest that anxiety led to a decreased influence of the goal-directed attentional system, which inturn led participant’s to be more likely to orientate attention away from goal-relevant information (i.e., the instruments crucial for performance of this task).

Partial evidence was found for anxiety leading to an increase in the randomness of gaze behavior. Specifically, whilst the ANOVA main effects for anxiety, or interactive effects of anxiety and cognitive load, on scanning entropy were not found, it was evident that the variation in response to the anxiety manipulation was most likely responsible for these null effects. Planned correlational analyses revealed that change in anxiety from the neutral to anxiety conditions correlated with change in scanning entropy, but interestingly, only when cognitive load was high. This result offers some support for the suggested (e.g., [ 4 ]) interaction between anxiety and working memory demands and is somewhat analogous to findings in simpler tasks [ 25, 26 ]. The ability to effectively seek important information in tasks adhering to an SSSI design philosophy may therefore be most impaired when both anxiety and cognitive load is high.

To elucidate support for the effects of anxiety on scanning randomness, we conducted a supplementary, alternative analysis, where participants were categorized into low- and high-anxiety manipulation responders. This analysis again revealed that entropy was higher for participants who had larger increases cognitive anxiety from neutral to anxiety conditions. When taken together, these results concur with previous studies [ 1, 37 ], and offers support for the suggestion that an individual’s reaction to a potentially anxiety-inducing situation is crucial in determining the effects on the predictability of gaze behavior. The interesting next step would be to determine exactly what underpins these anxiety-induced changes in scanning randomness. It is possible that the impairment to certain working memory functions (e.g., shifting, updating, inhibition) contributes to these changes.

The current study offers a number of interesting findings, however there are a number of limitations that should be considered when interpreting the results. Firstly, the participants in the current task were trained novices, therefore whether the various results generalize to other populations (i.e., true experts) requires further examination. Secondly, whilst the employed anxiety manipulation is readily used in perceptual-motor experiments, it cannot compare to real-world anxiety-inducing situations and is comparatively relatively weak. Thirdly, the sample size was relatively small, meaning that certain effects may not have been detected. Thirdly, adherence to the scan pattern specified during training may have somewhat dampened the effects of the independent variables on gaze behavior. Fourthly, subjective cognitive anxiety was measured after completion of each trial, potentially introducing retrospective bias. Specifically, participants may report higher anxiety after performing poorly.

While the study reported here is based on a flight control scenario, it holds broad implications for information visualization design. Visualizations can be dichotomized into those that are designed for “data availability” and those for “information extraction” [ 46 ]. It is often assumed that those that are designed to maximize “data availability” (e.g., SSSIs) burden the operator with the task of seeking out relevant data, maintaining this data in memory, while integrating these data to generate appropriate responses/decisions. The current results show that this is indeed the case. Visual scanning behavior across multiple and separate channels of information is an effortful process that is further compromised by operators’ states of anxiety and high working memory load. A shift to more integrated displays, such as ‘glass cockpits’, is not necessarily the solution, as they still present information in separate, albeit spatially closer, display regions. Indeed, there is some evidence to suggest that they can lead to poorer flight performance in novices [ 48 ]. It would be interesting for future research to explicitly examine whether differing data visualisation philosophies (e.g., SSSI compared to ecological interface design) differ in their capability for operators to use them in high anxiety, high cognitive load conditions.

### Ethics and Conflict of Interest

The author(s) declare(s) that the contents of the article are in agreement with the ethics described in http://biblio.unibe.ch/portale/elibrary/BOP/jemr/ethics.html and that there is no conflict of interest regarding the publication of this paper.

### Acknowledgements

This paper is an extension of ETVIS 2016 conference proceedings [ 2 ] and was based on work conducted during the first author's doctoral studies. We wish to thank Monika Marsching for her valuable input when designing the acquisition phase and flight task.

Jonathan Allsop was partly supported by a research grant from the Deutsche Akademische Austauschdienst. Lewis Chuang is supported by the German Research Foundation (DFG) within project C03 of SFB/Transregio 161

## Footnotes

When substituting heart rate for self-report cognitive anxiety data, a simi9 lar pattern of results was not found, changes in heart rate did not predict changes in scanning entropy in either low or high cognitive load conditions. However, while heart rate and cognitive anxiety are often somewhat correlated, it is argued that the former can also be indicative of general arousal. We thank an anonymous reviewer for this suggestion.
